# Dichlorido[2,2′-(oxydimethyl­ene)dipyridine]zinc(II)

**DOI:** 10.1107/S1600536808034600

**Published:** 2008-10-31

**Authors:** Jin Min Li

**Affiliations:** a Chemistry and Chemical Engineering College, Shanxi Datong University, Datong 037009, People’s Republic of China

## Abstract

In the title complex, [ZnCl_2_(C_12_H_12_N_2_O)], the Zn^II^ atom is coordinated in a distorted trigonal-bipyramidal geometry by two Cl atoms, and one O atom and two N atoms from the 2,2′-(oxydimethyl­ene)dipyridine ligand. In the complex, the two pyridine rings make a dihedral angle of 15.44 (14)°. There is a weak inter­molecular π–π stacking inter­action between pyridine rings; the centroid–centroid distance is 3.8079 (17) Å.

## Related literature

For the isotypic Cd and Cu analogs of the title compound, see: Li (2007 ▶) and Li (2008 ▶), respectively.
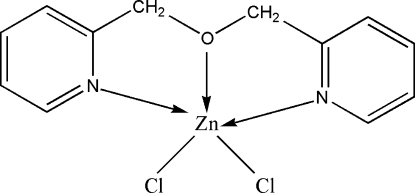


## Experimental

### 

#### Crystal data

[ZnCl_2_(C_12_H_12_N_2_O)]
*M* *_r_* = 336.51
Monoclinic, 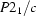

*a* = 8.0874 (12) Å
*b* = 12.5013 (18) Å
*c* = 15.6210 (16) Å
β = 121.180 (11)°
*V* = 1351.2 (3) Å^3^
*Z* = 4
Mo *K*α radiation
μ = 2.20 mm^−1^
*T* = 298 (2) K
0.40 × 0.32 × 0.13 mm

#### Data collection

Bruker SMART APEX CCD diffractometer
Absorption correction: multi-scan (**SADABS**; Sheldrick, 1996 ▶) *T*_min_ = 0.466, *T*_max_ = 0.751
5529 measured reflections
2388 independent reflections
2158 reflections with *I* > 2σ(*I*)
*R*_int_ = 0.024

#### Refinement

*R*[*F* ^2^ > 2σ(*F* ^2^)] = 0.026
*wR*(*F* ^2^) = 0.069
*S* = 1.05
2388 reflections
163 parameters
H-atom parameters constrained
Δρ_max_ = 0.51 e Å^−3^
Δρ_min_ = −0.35 e Å^−3^

### 

Data collection: *SMART* (Bruker, 1997 ▶); cell refinement: *SAINT* (Bruker, 1997 ▶); data reduction: *SAINT*; program(s) used to solve structure: *SHELXS97* (Sheldrick, 2008 ▶); program(s) used to refine structure: *SHELXL97* (Sheldrick, 2008 ▶); molecular graphics: *SHELXTL* (Sheldrick, 2008 ▶); software used to prepare material for publication: *SHELXTL*.

## Supplementary Material

Crystal structure: contains datablocks I, global. DOI: 10.1107/S1600536808034600/is2347sup1.cif

Structure factors: contains datablocks I. DOI: 10.1107/S1600536808034600/is2347Isup2.hkl

Additional supplementary materials:  crystallographic information; 3D view; checkCIF report

## Figures and Tables

**Table 1:** Selected bond lengths (Å)

Cl1—Zn1	2.2803 (6)
Cl2—Zn1	2.2642 (7)
N1—Zn1	2.1178 (18)
N2—Zn1	2.1128 (18)
O1—Zn1	2.2252 (16)

**Fig. 1.**
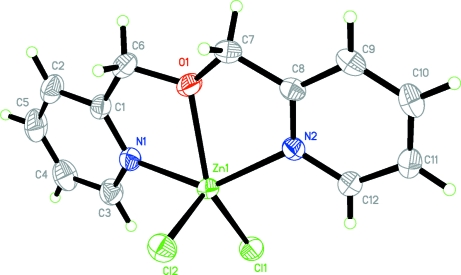
View of complex (I), showing the atom numbering scheme with thermal ellipsoids drawn at the 30% probability level

## supplementary crystallographic information

### Comment

2,2'-(oxydimethylene)dipyridine is an useful tridentate terminal ligand and the Cd^II^ complex with it as ligand has been published (Li, 2007). Herein the crystal structure of the title Zn complex, (I), with 2,2'-(oxydimethylene)dipyridine as ligand, is reported.

The molecular structure of (I) is shown in Fig. 1. In this mononuclear complex, atom Zn1 is in a distorted trigonal bipyramidal coordination environment (Table 1). In the crystal structure, there are a weak π-π stacking interaction between symmetry related pyridyl rings, with the relevant distances being *Cg*1···*Cg*1^i^ = 3.8079 (17) Å and a perpendicular distance of 3.597 Å [symmetry code (i) 1-*x*, 2-*y*, -*z*; *Cg*1 is the centroid of the N1/C1—C5 ring]. The title compound is isostructural with the Cd^II^ complex (Li, 2007).

### Experimental

A methanol solution (5 ml) of 2,2'-(oxydimethylene)dipyridine (0.0345 g, 0.172 mmol) was added into 8 ml H_2_O solution containing ZnCl_2_ (0.0241 g, 0.177 mmol). The mixed solution was stirred for a few minutes. The colorless single crystals were obtained after the solution had been allowed to stand at room temperature for two weeks.

### Refinement

All H atom were placed in calculated positions and refined as riding, with C—H = 0.93–0.97 Å, and with *U*_iso_(H) = 1.2*U*_eq_(C).

### Crystal data

**Table:**

[ZnCl_2_(C_12_H_12_N_2_O)]	*F*(000) = 680
*M**_r_* = 336.51	*D*_x_ = 1.654 Mg m^−^^3^
Monoclinic, *P*2_1_/*c*	Mo *K*α radiation, λ = 0.71073 Å
Hall symbol: -P 2ybc	Cell parameters from 3915 reflections
*a* = 8.0874 (12) Å	θ = 2.9–28.1°
*b* = 12.5013 (18) Å	µ = 2.20 mm^−^^1^
*c* = 15.6210 (16) Å	*T* = 298 K
β = 121.180 (11)°	Block, colorless
*V* = 1351.2 (3) Å^3^	0.40 × 0.32 × 0.13 mm
*Z* = 4	

### Data collection

**Table:**

Bruker SMART APEX CCD diffractometer	2388 independent reflections
Radiation source: fine-focus sealed tube	2158 reflections with *I* > 2σ(*I*)
graphite	*R*_int_ = 0.024
φ and ω scans	θ_max_ = 25.0°, θ_min_ = 2.2°
Absorption correction: multi-scan (*SADABS*; Sheldrick, 1996)	*h* = −8→9
*T*_min_ = 0.466, *T*_max_ = 0.751	*k* = −14→8
5529 measured reflections	*l* = −17→18

### Refinement

**Table:**

Refinement on *F*^2^	Primary atom site location: structure-invariant direct methods
Least-squares matrix: full	Secondary atom site location: difference Fourier map
*R*[*F*^2^ > 2σ(*F*^2^)] = 0.026	Hydrogen site location: inferred from neighbouring sites
*wR*(*F*^2^) = 0.069	H-atom parameters constrained
*S* = 1.05	*w* = 1/[σ^2^(*F*_o_^2^) + (0.0373*P*)^2^ + 0.2883*P*] where *P* = (*F*_o_^2^ + 2*F*_c_^2^)/3
2388 reflections	(Δ/σ)_max_ = 0.001
163 parameters	Δρ_max_ = 0.51 e Å^−^^3^
0 restraints	Δρ_min_ = −0.35 e Å^−^^3^

### Special details

**Table:**

Geometry. All e.s.d.'s (except the e.s.d. in the dihedral angle between two l.s. planes) are estimated using the full covariance matrix. The cell e.s.d.'s are taken into account individually in the estimation of e.s.d.'s in distances, angles and torsion angles; correlations between e.s.d.'s in cell parameters are only used when they are defined by crystal symmetry. An approximate (isotropic) treatment of cell e.s.d.'s is used for estimating e.s.d.'s involving l.s. planes.
Refinement. Refinement of *F*^2^ against ALL reflections. The weighted *R*-factor *wR* and goodness of fit *S* are based on *F*^2^, conventional *R*-factors *R* are based on *F*, with *F* set to zero for negative *F*^2^. The threshold expression of *F*^2^ > σ(*F*^2^) is used only for calculating *R*-factors(gt) *etc*. and is not relevant to the choice of reflections for refinement. *R*-factors based on *F*^2^ are statistically about twice as large as those based on *F*, and *R*- factors based on ALL data will be even larger.

### Fractional atomic coordinates and isotropic or equivalent isotropic displacement parameters (Å^2^)

**Table:**

	*x*	*y*	*z*	*U*_iso_*/*U*_eq_	
C1	0.4107 (3)	0.83139 (18)	−0.04103 (18)	0.0431 (5)	
C2	0.2717 (3)	0.9110 (2)	−0.0806 (2)	0.0609 (7)	
H2	0.2077	0.9271	−0.1485	0.073*	
C3	0.4597 (4)	0.8595 (2)	0.1155 (2)	0.0560 (6)	
H3	0.5236	0.8417	0.1831	0.067*	
C4	0.3238 (4)	0.9395 (2)	0.0809 (3)	0.0687 (8)	
H4	0.2964	0.9752	0.1243	0.082*	
C5	0.2299 (4)	0.9658 (2)	−0.0178 (3)	0.0698 (8)	
H5	0.1384	1.0203	−0.0425	0.084*	
C6	0.4609 (3)	0.7685 (2)	−0.10595 (18)	0.0503 (6)	
H6A	0.3458	0.7537	−0.1702	0.060*	
H6B	0.5515	0.8079	−0.1171	0.060*	
C7	0.6087 (3)	0.59950 (19)	−0.09923 (16)	0.0460 (5)	
H7A	0.6875	0.6357	−0.1205	0.055*	
H7B	0.4990	0.5673	−0.1575	0.055*	
C8	0.7246 (3)	0.51540 (18)	−0.02358 (16)	0.0397 (5)	
C9	0.7807 (3)	0.4226 (2)	−0.05024 (19)	0.0495 (6)	
H9	0.7444	0.4107	−0.1165	0.059*	
C10	0.8902 (4)	0.3486 (2)	0.0219 (2)	0.0537 (6)	
H10	0.9305	0.2863	0.0054	0.064*	
C11	0.9394 (3)	0.36760 (19)	0.1189 (2)	0.0508 (6)	
H11	1.0125	0.3182	0.1690	0.061*	
C12	0.8788 (3)	0.46096 (18)	0.14068 (18)	0.0456 (5)	
H12	0.9123	0.4736	0.2064	0.055*	
Cl1	0.71768 (8)	0.63978 (5)	0.25220 (4)	0.04925 (16)	
Cl2	0.98849 (8)	0.77735 (5)	0.14453 (5)	0.05218 (16)	
N1	0.5035 (3)	0.80611 (14)	0.05554 (14)	0.0426 (4)	
N2	0.7730 (2)	0.53478 (14)	0.07069 (13)	0.0386 (4)	
O1	0.5448 (3)	0.67253 (13)	−0.05431 (12)	0.0539 (4)	
Zn1	0.71979 (3)	0.686250 (19)	0.111610 (17)	0.03705 (11)	

### Atomic displacement parameters (Å^2^)

**Table:**

	*U*^11^	*U*^22^	*U*^33^	*U*^12^	*U*^13^	*U*^23^
C1	0.0374 (11)	0.0391 (12)	0.0482 (14)	−0.0003 (9)	0.0189 (10)	0.0079 (10)
C2	0.0492 (14)	0.0550 (15)	0.0680 (17)	0.0116 (12)	0.0229 (13)	0.0196 (14)
C3	0.0644 (15)	0.0483 (14)	0.0592 (16)	0.0069 (12)	0.0346 (13)	−0.0003 (12)
C4	0.0747 (18)	0.0504 (16)	0.094 (2)	0.0070 (14)	0.0533 (18)	−0.0080 (16)
C5	0.0595 (16)	0.0487 (15)	0.104 (2)	0.0156 (13)	0.0439 (17)	0.0120 (16)
C6	0.0497 (13)	0.0538 (14)	0.0417 (13)	0.0048 (11)	0.0196 (10)	0.0125 (11)
C7	0.0496 (12)	0.0524 (14)	0.0371 (12)	−0.0020 (11)	0.0232 (10)	−0.0055 (11)
C8	0.0393 (11)	0.0425 (12)	0.0393 (12)	−0.0079 (9)	0.0217 (9)	−0.0068 (10)
C9	0.0547 (13)	0.0495 (14)	0.0519 (14)	−0.0101 (11)	0.0329 (12)	−0.0153 (12)
C10	0.0568 (14)	0.0395 (12)	0.0784 (19)	−0.0017 (11)	0.0444 (14)	−0.0067 (13)
C11	0.0491 (13)	0.0425 (13)	0.0603 (16)	0.0047 (10)	0.0281 (12)	0.0069 (11)
C12	0.0471 (12)	0.0437 (13)	0.0430 (13)	0.0021 (10)	0.0213 (10)	0.0012 (10)
Cl1	0.0624 (3)	0.0501 (3)	0.0403 (3)	−0.0039 (3)	0.0301 (3)	0.0019 (3)
Cl2	0.0507 (3)	0.0541 (3)	0.0561 (4)	−0.0081 (3)	0.0307 (3)	−0.0034 (3)
N1	0.0420 (10)	0.0387 (10)	0.0445 (11)	0.0025 (8)	0.0206 (9)	0.0011 (8)
N2	0.0419 (9)	0.0362 (9)	0.0380 (10)	−0.0004 (7)	0.0208 (8)	−0.0025 (8)
O1	0.0673 (10)	0.0512 (10)	0.0332 (9)	0.0166 (8)	0.0190 (8)	0.0023 (7)
Zn1	0.04082 (16)	0.03641 (17)	0.03043 (17)	0.00126 (10)	0.01599 (12)	−0.00073 (10)

### Geometric parameters (Å, °)

**Table:**

C1—N1	1.329 (3)	C7—H7B	0.9700
C1—C2	1.384 (3)	C8—N2	1.336 (3)
C1—C6	1.496 (3)	C8—C9	1.386 (3)
C2—C5	1.375 (4)	C9—C10	1.370 (4)
C2—H2	0.9300	C9—H9	0.9300
C3—N1	1.339 (3)	C10—C11	1.373 (4)
C3—C4	1.373 (4)	C10—H10	0.9300
C3—H3	0.9300	C11—C12	1.375 (3)
C4—C5	1.360 (4)	C11—H11	0.9300
C4—H4	0.9300	C12—N2	1.346 (3)
C5—H5	0.9300	C12—H12	0.9300
C6—O1	1.409 (3)	Cl1—Zn1	2.2803 (6)
C6—H6A	0.9700	Cl2—Zn1	2.2642 (7)
C6—H6B	0.9700	N1—Zn1	2.1178 (18)
C7—O1	1.403 (3)	N2—Zn1	2.1128 (18)
C7—C8	1.494 (3)	O1—Zn1	2.2252 (16)
C7—H7A	0.9700		
			
N1—C1—C2	121.9 (2)	C10—C9—H9	120.3
N1—C1—C6	116.90 (19)	C8—C9—H9	120.3
C2—C1—C6	121.2 (2)	C9—C10—C11	119.0 (2)
C5—C2—C1	118.8 (3)	C9—C10—H10	120.5
C5—C2—H2	120.6	C11—C10—H10	120.5
C1—C2—H2	120.6	C10—C11—C12	118.9 (2)
N1—C3—C4	122.4 (3)	C10—C11—H11	120.5
N1—C3—H3	118.8	C12—C11—H11	120.5
C4—C3—H3	118.8	N2—C12—C11	122.6 (2)
C5—C4—C3	119.1 (3)	N2—C12—H12	118.7
C5—C4—H4	120.5	C11—C12—H12	118.7
C3—C4—H4	120.5	C1—N1—C3	118.5 (2)
C4—C5—C2	119.3 (2)	C1—N1—Zn1	119.86 (15)
C4—C5—H5	120.4	C3—N1—Zn1	121.64 (17)
C2—C5—H5	120.4	C8—N2—C12	118.13 (19)
O1—C6—C1	106.18 (18)	C8—N2—Zn1	120.46 (14)
O1—C6—H6A	110.5	C12—N2—Zn1	120.81 (15)
C1—C6—H6A	110.5	C7—O1—C6	117.43 (18)
O1—C6—H6B	110.5	C7—O1—Zn1	117.11 (13)
C1—C6—H6B	110.5	C6—O1—Zn1	115.38 (14)
H6A—C6—H6B	108.7	N2—Zn1—N1	139.71 (7)
O1—C7—C8	107.76 (17)	N2—Zn1—O1	71.63 (6)
O1—C7—H7A	110.2	N1—Zn1—O1	71.44 (7)
C8—C7—H7A	110.2	N2—Zn1—Cl2	101.41 (5)
O1—C7—H7B	110.2	N1—Zn1—Cl2	103.18 (5)
C8—C7—H7B	110.2	O1—Zn1—Cl2	105.09 (5)
H7A—C7—H7B	108.5	N2—Zn1—Cl1	99.63 (5)
N2—C8—C9	121.9 (2)	N1—Zn1—Cl1	99.33 (6)
N2—C8—C7	116.73 (19)	O1—Zn1—Cl1	141.69 (5)
C9—C8—C7	121.4 (2)	Cl2—Zn1—Cl1	113.22 (3)
C10—C9—C8	119.4 (2)		
			
N1—C1—C2—C5	0.5 (4)	C8—C7—O1—Zn1	25.3 (2)
C6—C1—C2—C5	179.5 (2)	C1—C6—O1—C7	−178.79 (18)
N1—C3—C4—C5	0.0 (4)	C1—C6—O1—Zn1	−34.2 (2)
C3—C4—C5—C2	0.7 (4)	C8—N2—Zn1—N1	40.8 (2)
C1—C2—C5—C4	−0.9 (4)	C12—N2—Zn1—N1	−148.24 (15)
N1—C1—C6—O1	20.9 (3)	C8—N2—Zn1—O1	16.36 (15)
C2—C1—C6—O1	−158.1 (2)	C12—N2—Zn1—O1	−172.68 (17)
O1—C7—C8—N2	−11.5 (3)	C8—N2—Zn1—Cl2	−85.92 (15)
O1—C7—C8—C9	169.19 (19)	C12—N2—Zn1—Cl2	85.04 (16)
N2—C8—C9—C10	−0.5 (3)	C8—N2—Zn1—Cl1	157.83 (14)
C7—C8—C9—C10	178.7 (2)	C12—N2—Zn1—Cl1	−31.21 (16)
C8—C9—C10—C11	0.9 (3)	C1—N1—Zn1—N2	−40.5 (2)
C9—C10—C11—C12	−0.6 (3)	C3—N1—Zn1—N2	140.93 (17)
C10—C11—C12—N2	−0.1 (4)	C1—N1—Zn1—O1	−16.06 (16)
C2—C1—N1—C3	0.2 (3)	C3—N1—Zn1—O1	165.40 (19)
C6—C1—N1—C3	−178.8 (2)	C1—N1—Zn1—Cl2	85.67 (16)
C2—C1—N1—Zn1	−178.39 (17)	C3—N1—Zn1—Cl2	−92.87 (18)
C6—C1—N1—Zn1	2.6 (3)	C1—N1—Zn1—Cl1	−157.66 (16)
C4—C3—N1—C1	−0.5 (4)	C3—N1—Zn1—Cl1	23.80 (18)
C4—C3—N1—Zn1	178.1 (2)	C7—O1—Zn1—N2	−23.24 (15)
C9—C8—N2—C12	−0.2 (3)	C6—O1—Zn1—N2	−167.91 (17)
C7—C8—N2—C12	−179.44 (19)	C7—O1—Zn1—N1	173.15 (17)
C9—C8—N2—Zn1	171.03 (15)	C6—O1—Zn1—N1	28.48 (15)
C7—C8—N2—Zn1	−8.2 (2)	C7—O1—Zn1—Cl2	74.03 (16)
C11—C12—N2—C8	0.5 (3)	C6—O1—Zn1—Cl2	−70.65 (16)
C11—C12—N2—Zn1	−170.71 (17)	C7—O1—Zn1—Cl1	−105.42 (16)
C8—C7—O1—C6	169.28 (19)	C6—O1—Zn1—Cl1	109.91 (15)
